# The efficacy of L-carnitine in improving malnutrition in patients on maintenance hemodialysis: a meta-analysis

**DOI:** 10.1042/BSR20201639

**Published:** 2020-06-15

**Authors:** Jianwei Zhou, Tubao Yang

**Affiliations:** 1 Department of Epidemiology and Health Statistics, Xiangya School of Public Health, Central South University, Changsha 410078, Hunan Province, China; 2 Department of Nursing, Yueyang Second People's Hospital, Yueyang 414000, Hunan Province, China

**Keywords:** L-carnitine, maintenance hemodialysis, malnutrition, meta-analysis

## Abstract

The improvement of malnutrition with levocarnitine in maintenance hemodialysis (MHD) patients is controversial. We performed a meta-analysis to evaluate the efficacy of levocarnitine in improving malnutrition in MHD patients. We performed a literature search for relevant articles related to the treatment of malnutrition by L-carnitine in MHD patients in PubMed, Embase, Web of Science, China National Knowledge Infrastructure, and Wanfang databases. We set the publication dates from 1950 to July 2019. The levels of albumin, prealbumin, total protein, and transferrin before and after treatment were used for assessing malnutrition. Twenty-seven studies were included in the present analysis. The results of the random effects model indicated that L-carnitine treatment improved the albumin level in patients on MHD patients. The pooled standardized mean difference of albumin level was 2.51 (95% confidence interval (CI): 2.13−2.90, *P*<0.001). The pooled total protein level was 3.83 (95% CI: 2.41−5.24, *P* = 0.000) and the pooled transferrin level was 0.35 (95% CI: 0.18−0.52, *P* = 0.000). Significant differences were observed with the total protein and transferrin levels. The results indicated that levocarnitine significantly improved the prealbumin level in patients on MHD. The pooled prealbumin level was 70.86 (95% CI: 42.99−98.73, *P* = 0.000). No publication bias was detected (*P*>0.05). The present meta-analysis indicated that L-carnitine can have a favorable effect on malnutrition biomarkers in patients on MHD, including the increase in albumin, total protein, transferrin, and prealbumin levels. The L-carnitine could be an option for treatment of MHD patients.

## Introduction

As the prevalence of chronic kidney disease (CKD) increases every year, the prevalence of end-stage renal disease also increases [1]. Currently, maintenance hemodialysis (MHD) has become the mainstay of treatment in patients with end-stage renal disease [2]. The number of patients on global MHD is increasing rapidly, with the number of patients on dialysis increasing by 6% each year. It is estimated that more than 2.2 million patients will undergo dialysis globally by 2020 [3].

Malnutrition is a common complication of MHD. The incidence of malnutrition in patients undergoing MHD was reported to be 15−75%, of which about 6−8% of the patients had severe malnutrition [4]. Studies have shown that malnutrition is one of the main factors affecting the survival of patients undergoing dialysis [5]. Traditionally, the concept of malnutrition in CKD dialysis patients usually refers to nutritional abnormality caused by insufficient dietary protein intake or excessive loss of albumin that was once referred to as ‘malnutrition inflammation complex syndrome’ or ‘uremic malnutrition’ [6]. In 2010, the International Society of Renal Nutrition and Metabolism has highlighted a reduced protein and energy reserve associated with the progress of CKD [7]. The clinical signs of adequate nutrition and calorie intake, low body mass index (BMI), low serum albumin level, presence of micro-inflammation, and progressive skeletal muscle consumption are used to diagnose malnutrition syndrome, also known as protein-energy wasting (PEW) [8]. The diagnostic criteria include a biochemical index, BMI, muscle loss, and diet [9]. The mechanism of the development of PEW is complex. Currently, it is believed that the main causes of PEW in patients undergoing MHD include malnutrition, anorexia, gastrointestinal dysfunctions, endocrine disorders, metabolic acidosis, insulin resistance, disorders related to amino acids and minerals, elevated leptin level, inflammation, and dialysis-associated protein and nutrient loss in CKD [10]. Malnutrition not only reduces the quality of life of the patients but also affects the survival rate and risk factors of mortality [11]. At present, the nutritional indexes of MHD patients include the following: levels of serum albumin, prealbumin, hemoglobin, total cholesterol, uric acid, creatinine, blood urea nitrogen, transferrin, and insulin-like growth factor 1; the total number of lymphocytes; etc. [12]. Among these, serum albumin level is the most important and commonly used biochemical index to reflect visceral protein storage, which is not only an indicator of malnutrition in dialysis patients but also a sensitive indicator to judge the mortality rate of patients on dialysis [13].

Levo (L)-carnitine (L-CN), also known as carnitine or carnitine and vitamin BT, is a natural substance necessary for energy metabolism in the mammals. L-carnitine is a special amino acid necessary for fat metabolism in the body [14]. Due to reduced intake and rapid clearance by dialysis, patients on MHD generally have a deficiency of L-CN, thereby aggravating several complications, such as dialysis hypotension, myocardial damage, malnutrition, and anemia [15]. However, the improvement of malnutrition with levocarnitine in patients on MHD is controversial. While some studies suggest that L-CN can improve malnutrition in MHD patients [16], others mention that it does not [17]. Therefore, we performed a meta-analysis to assess the efficacy of levocarnitine in improving malnutrition in MHD patients.

## Material and methods

We performed this meta-analysis by following the Preferred Reporting Items for Systematic Reviews and Meta-analysis [18].

### Search strategy

We performed a literature search in PubMed, Embase, Web of Science, China National Knowledge Infrastructure, and Wanfang databases. We set the publication dates from 1950 to May 25, 2020. The following combination of search terms were used: ‘carnitine’, ‘L-carnitine’, ‘levocarnitine’, ‘maintenance hemodialysis’, ‘hemodialysis’, ‘MHD’, ‘malnutrition’, ‘nutrition’, ‘randomized controlled trial’, ‘controlled clinical trial’, ‘randomized’, ‘controlled’, ‘trial’, ‘placebo’, and ‘randomized controlled trial (RCT)’. We restricted the search language to Chinese and English. We also reviewed the reference lists of the relevant review articles for potential studies. If necessary, we tried to contact the author to obtain the undisclosed data. The search strategy with the search details is presented in the supplementary file (Supplementary material 1). The research has been carried out in accordance with the World Medical Association Declaration of Helsinki, and that all subjects provided informed consent.

### Inclusion and exclusion criteria

The studies were considered eligible if (1) the study population was of ≥18 years of age and without any infections, trauma, tumors, or gastrointestinal tract diseases; (2) patients underwent hemodialysis for at least 6 months; (3) the study was a randomized controlled trial or a prospective cohort study; (4) one group received levocarnitine (20 mg/kg) and the control group received placebo or blank control or usual treatment; (5) the language was Chinese or English; and (6) the study had one or more outcomes as follows: albumin (Alb), total protein (TP), transferrin (TRF), and prealbumin (PA) levels. The exclusion criteria were: (1) patients on non-MHD; (2) no available data or incomplete data for pooling; (3) duplicates data or studies; (4) animal experiments, reviews, comments, letters, editorials, or case reports.

### Data extraction

We used a data abstraction form and extracted the following data: the surname of the first author, publication year, mean age or age range of the trial and the control groups, sample size, the number of male and female participants, the type of disease, intervention used in the trial and the control groups, and outcomes, including TP, Alb, TRF, and PA levels. We also checked the extracted data for accuracy. The inconsistencies were resolved by consensus.

### Assessment of quality

The Cochrane risk of bias tool was used to assess the quality of the included studies [19]. This tool consists of the following primary items: random sequence generation, allocation concealment, blinding of participants, personnel to study protocol, and blinding of outcome assessment, incomplete outcomes data, selective reporting, and other bias. We judged the study as of low risk, unclear risk, and high risk by evaluating the items. Studies with more than one primary item were considered as having high risks of bias, while those without all the domains were considered as having low risks of bias. Otherwise, the studies were of unclear risk of bias. We used the summary plot to present the results of the quality assessment.

### Statistical analysis

Because some of the outcome variables were continuous and the units in each variable were consistent across the included studies, we used the weighted mean difference (WMD) to pool the data and estimate the 95% confidence interval (CI) [20]. For albumin level, the unit was different across various studies, and hence, the standardized mean difference (SMD) was used, and WMD will be used for parameter with the same unit. [21]. The heterogeneity within the studies was assessed by *I*^2^ statistics and Cochran's *Q*-test. A *P*-value of <0.10 for Cochran's *Q*-test or an *I*^2^ value of >50% was considered as the presence of significant heterogeneity. We pooled the results using the random effects model [22]. Otherwise, we selected the fixed effects model [23]. Sensitivity analysis was subsequently performed to substantiate the robustness of the synthetic results and identify the influential studies. Finally, we performed Begg's test and Egger's test to evaluate the presence of potential publication bias among the studies, and a funnel plot was constructed for more than 10 studies [24]. A *P*-value of <0.05 was considered statistically significant. All analyses were performed with STATA version 14.0.

## Results

### Study selection and study characteristics

**Figure 1:**
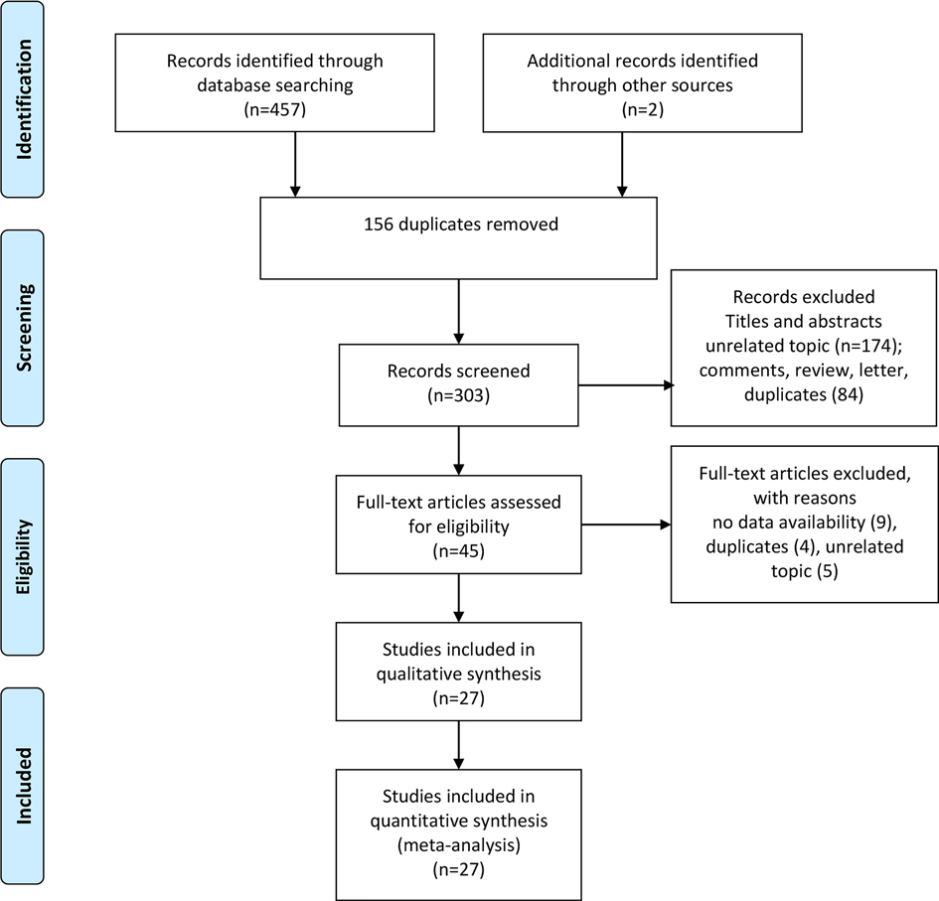
The flow chart of study selection

Our initial search returned 459 records from all the databases. Two studies were identified from the references of the relevant articles. Three hundred and three records were further screened by titles and abstracts and 156 duplicate articles were removed. We excluded unrelated articles, comments, reviews, letters, and duplicate articles. Then 45 articles were assessed by accessing the full-text. Eighteen studies were excluded because of unavailable data, duplicates, and unrelated topics. Finally, 27 studies were included in the analysis (supplementary material 2). Figure 1 shows a flowchart of the study selection process.

Twenty-seven studies were published from 1990 to 2017. The total sample size was 1682, including 844 trials and 838 controls. The sample size ranged from 32 to 127. There were 934 male and 748 female patients. These studies were from different countries including China (*n* = 22), U.S.A. (*n* = 2), Turkey (*n* = 2), and Italy (*n* = 2). The mean age of the trial group was almost equal to that of the control group. The primary disease types were – I: hypertensive nephropathy, II: diabetic nephropathy, III: chronic glomerulonephritis, IV: obstructive nephropathy, V: polycystic kidney, VI: uric acid nephropathy, VII: chronic pyelonephritis; VIII: gout nephropathy; IX: chronic interstitial nephritis, and X: lupus nephritis. The study population in the trial group received L-carnitine. As a control, 8 studies used a placebo, 12 used blank (no treatment), one used α-ketoacid, and one used routine treatment. Twenty-two studies reported albumin levels, 8 reported total protein levels, 5 reported transferrin levels, and 11 reported pre-albumin levels. Table 1 depicts the detailed general characteristics of the included studies.

**Table 1:** General characteristics of included studies in the meta-analysis

Author	Year	Country	Trial age	Control age	Sample size	Male	Female	Type of diseases	Dose	Intervention		Outcomes
										Trial group	Control	
Murat	2006	Turkey	44.0±13.9	43.4±13.9	42	24	18	I, II, III, IV	20 mg/kg	L-carnitine	No L-CN	TP, Alb, TRF
Xu	2017	China	53.2±1.2	52.9±1.1	100	62	38	XI	10–20 mg/kg	L-carnitine	α-Ketoacid	TP, Alb, TRF
Chen	2014	China	53.4±3.1	52.9±2.9	40	22	18	I, II, III, VI	1 g*	L-carnitine	Placebo	Alb, PA
Jin	2011	China	57.6±10.6	55.7±12.7	39	23	16	XI	1 g	L-carnitine	Placebo	Alb, PA
Xue	2013	China	69.6±5.4	69.6±5.4	46	29	17	XI	1 g	L-carnitine	No L-CN	Alb, PA
Qin	2014	China	71.2±10.4	70.9±9.7	54	24	30	XI	1 g	L-carnitine	No L-CN	TP, Alb, PA
Wang	2017	China	26-55	25-65	88	51	37	I, II, III, V	1 g	L-carnitine	Placebo	Alb, PA
Guo	2014	China	62.5±11.0	61.7±11.6	40	26	14	III, V, VII, VIII, IX	1 g	L-carnitine	No L-CN	Alb, PA
Zhu	2012	China	52.9±16.4	52.8±14.0	40	33	17	I, II, III, IV, IX, X	1 g	L-carnitine	No L-CN	TP, Alb, PA
Lu	2014	China	52.7±7.5	52.3±7.5	138	75	63	XI	1 g	L-carnitine	Placebo	TP, Alb, PA
Fan	2009	China	20-65	20-65	32	20	12	XI	1 g	L-carnitine	Placebo	Alb
Ma	2013	China	61.3±9.6	60.7±11.2	68	41	27	I, II, III, V	1 g	L-carnitine	No L-CN	Alb
Yu	2016	China	55.2±19.8	56.4±18.5	62	32	30	I, II, III, X	1 g	L-carnitine	No L-CN	Alb
Pan	2012	China	45.6±9.4	45.6±9.4	58	29	29	XI	1 g	L-carnitine	No L-CN	TP, Alb, PA
Li	2012	China	25-83	30-89	50	26	24	I, II, III, VIII	1 g	L-carnitine	Routine	Alb, PA
Lin	2015	China	58.9±9.1	59.3±9.4	62	37	26	I, II, III, V	1 g	L-carnitine	Placebo	Alb
Sun	2017	China	71.5±6.1	70.6±6.2	71	39	32	I, II, III, X	1 g	L-carnitine	No L-CN	Alb, PA
Tian	2011	China	45.3±17.8	44.8±17.3	50	31	19	I, II, III, V, VIII	1 g	L-carnitine	Placebo	Alb
Zhang	2009	China	45.8±14.3	15.8±14.3	65	35	30	I, II, III, V, X	1 g	L-carnitine	No L-CN	TP, Alb, TRF
Liu	2012	China	41.9±4.8	42.1±4.9	52	31	25	I, II, III, V, X, XI	1 g	L-carnitine	No L-CN	TP, Alb, TRF
Yao	2007	China	49.6±13.2	49.6±13.2	78	41	37	I, II, III, V, VII, X	1 g	L-carnitine	Placebo	TP, Alb, TRF
Ran	2012	China	63.2±11.4	63.2±11.4	127	71	56	II, III, V, VII	1 g	L-carnitine	No L-CN	Alb, TRF, PA
Ahmad	1990	U.S.A.	–	–	82	42	40	I, VII	20 mg/kg	L-carnitine	Placebo	Alb
Biolo	2008	Italy	63.0±3	57±4	19	44	8	I, V, VII	20 mg/kg	L-carnitine	Placebo	Alb
Duranav	2006	Turkey	44.0±13.9	43.4±13.9	42	12	30	I, V, VII	20 mg/kg	L-carnitine	Placebo	Alb
Savica	2005	Italy	63.3±16.5	61.1±12.5	103	29	72	I, III, VII	20 mg/kg	L-carnitine	Placebo	Alb
Steiber	2006	U.S.A.	67.6±3.9	69.4±3.4	34	11	23	III, V, VII	20 mg/kg	L-carnitine	Placebo	Alb

I: hypertensive nephropathy, II: diabetic nephropathy, III: chronic glomerulonephritis, IV: obstructive nephropathy, V: polycystic kidney, VI: uric acid nephropathy, VII: chronic pyelonephritis; VIII: gout nephropathy; IX: chronic interstitial nephritis, X: lupus nephritis, XI: not report.

* Intravenous injection 1 g after each treatment.

### Assessment of quality

The quality assessment is represented in Supplementary Figure S3 (Supplementary material 3) and Supplementary Figure S4 (Supplementary material 4). Overall, 12 studies were categorized as having high risks, and the rest of the studies had unclear risks of bias. The primary reason for the high risk was that these studies did not report whether the participants and personnel were blinded (performance bias). The method of random sequence generation was perfect in all the studies.

### Pooled results

#### Albumin level

**Figure 2:**
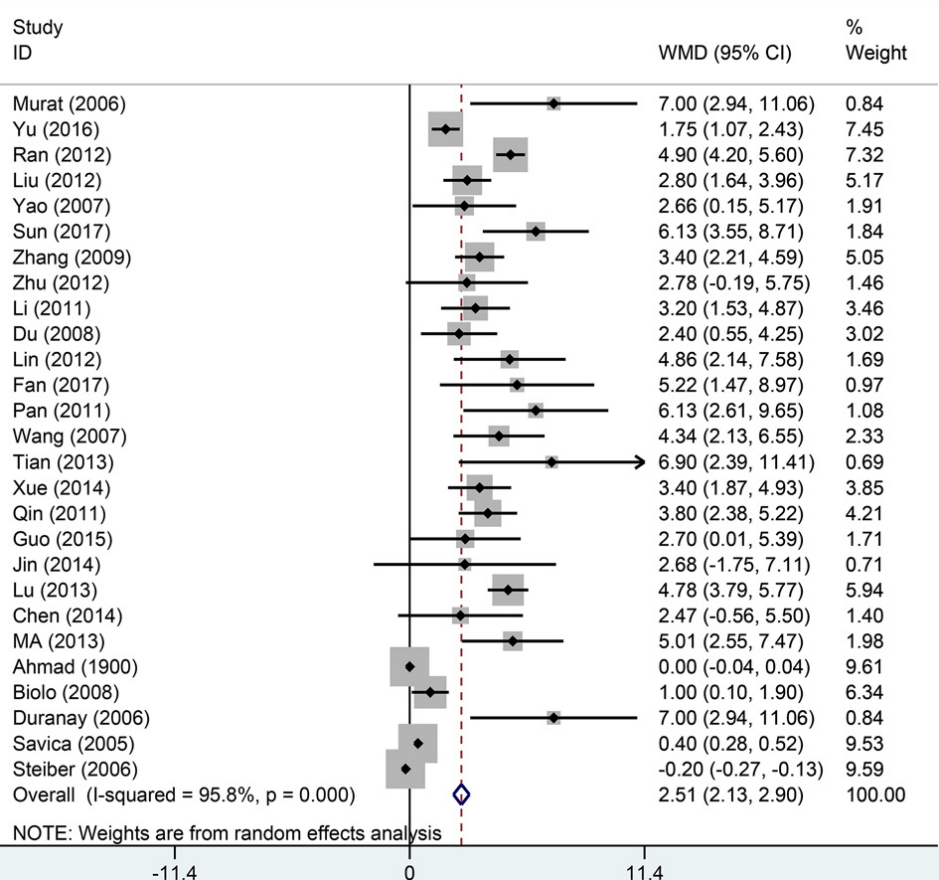
Forest plot of studies comparing the effect of L-carnitine versus control on serum albumin in hemodialysis patients

Twenty-seven studies provided the albumin level. The *I*^2^ and *Q*-test indicated the existence of a moderate heterogeneity (*I*^2^ = 81.8%, *P*<0.001). The random effects model was used. The results indicated the L-carnitine treatment improved the albumin level in patients on MHD. The pooled SMD of albumin was 2.51 (95% CI: 2.13−2.90, *P*<0.001, Figure 2). A subgroup analysis was conducted by excluding several studies having a potential high risk. Type I: we excluded two studies which included patients with a mean age of >70 years. The pooled WMD of albumin level was 3.52 (95% CI: 3.18−3.84, *P*<0.001) using the random effects model and the heterogeneity was moderate. We divided all the studies into placebo and blank control groups. The heterogeneity within the studies was moderate and the pooled results were 3.96 (95% CI: 3.05−3.75) and 3.58 (95% CI: 3.23−3.94), respectively. The results of the subgroup analysis were consistent with that of the total pooled result. The detailed results are presented in Table 2.

**Table 2:** Summary of pooled results in the meta-analysis

Category	Pooled results	95%CI	*Z*	*P* *	*I^2^* (%)	Egger	Begg
Albumin	2.51	2.13–2.90	7.25	*P*<0.001	81.4	0.282	0.338
Type 1	3.52	3.18–3.84	20.76	*P*<0.001	62.9	0.400	0.284
Type 2	3.96	3.05–3.75	18.99	*P*<0.001	55.3	0.282	0.198
Type 3	3.58	3.23–3.94	19.84	*P*<0.001	60.2	0.370	0.444
Type 4	1.32	0.80–1.23	8.890	*P*<0.001	75.5	0.494	0.573
Total protein	3.83	2.41–5.24	6.20	*P*<0.001	9.7	0.108	0.213
Transferrin	0.35	0.18–0.52	5.73	*P*<0.001	33.1	0.639	0.462
Prealbumin	70.86	42.99–98.73	14.48	*P*<0.001	96.8	0.082	0.087

*Heterogeneity.

Type 1: excluded two studies with patients over 70 years old (mean).

Type 2: only placebo.

Type 3: only control.

Type 4: excluded five studies with less than 40 patients.

#### Total protein and transferrin levels

**Figure 3:**
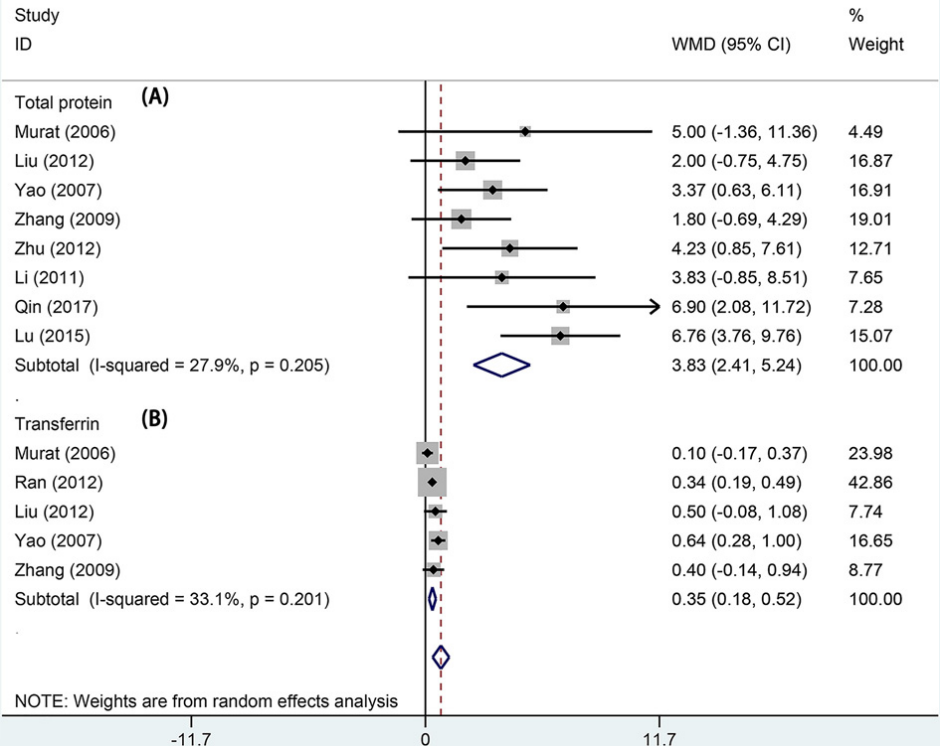
Forest plot of studies comparing the effect of L-carnitine versus control on nutribution of hemodialysis patients Forest plot of studies comparing the effect of L-carnitine versus control on total protein (**A**) and transferrin (**B**) in hemodialysis patients

Eight studies were included for pooling the total protein level. No significant heterogeneity existed within the studies (*I*^2^ = 27.9%, *P* = 0.205). The results of the fixed effects model indicated that levocarnitine treatment could improve the total protein level in patients on MHD. The pooled total protein level was 3.83 (95% CI: 2.41−5.24, *P*<0.001, Figure 3A). Five studies presented the results of transferrin level. There was no significant heterogeneity within the studies (*I*^2^ = 33.1%, *P* = 0.201). We used the fixed effects model to pool the data. The pooled transferrin level was 0.35 (95% CI: 0.18−0.52, *P*<0.001, Figure 3B). Significant differences were observed between the total protein and transferrin levels.

#### Prealbumin level

**Figure 4:**
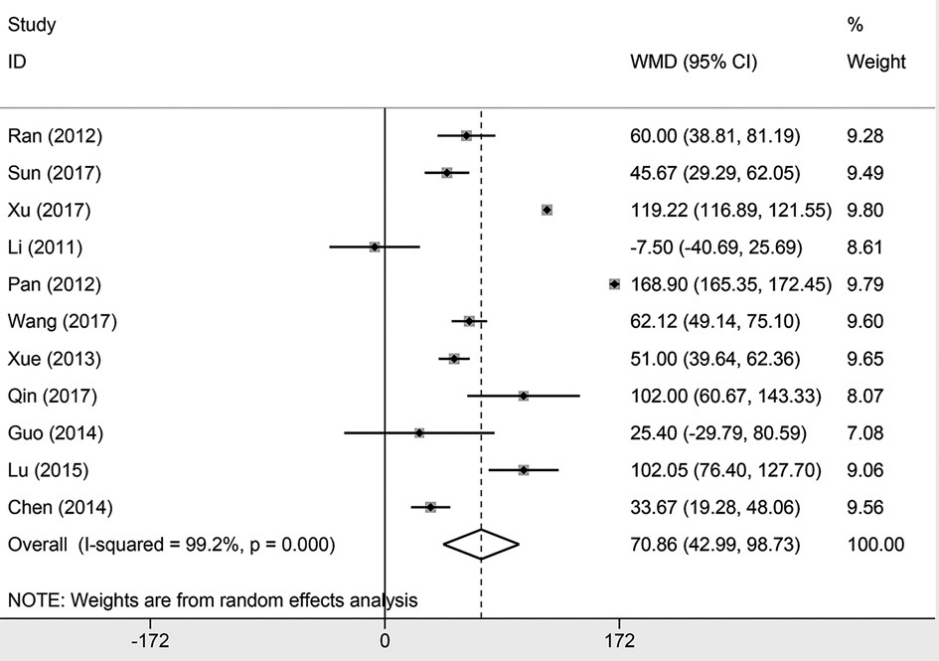
Forest plot of studies comparing the effect of L-carnitine versus control on prealbumin in hemodialysis patients

Eleven studies presented the results of prealbumin level. There was significant heterogeneity within the studies (*I*^2^ = 99.2%, *P*<0.001). We used the random effects model to pool the results. The results indicated that levocarnitine could significantly improve the prealbumin level in patients on MHD. The pooled prealbumin level was 70.86 (95% CI: 42.99−98.73, *P*<0.001, Figure 4).

### Sensitivity analysis and publication bias

**Figure 5:**
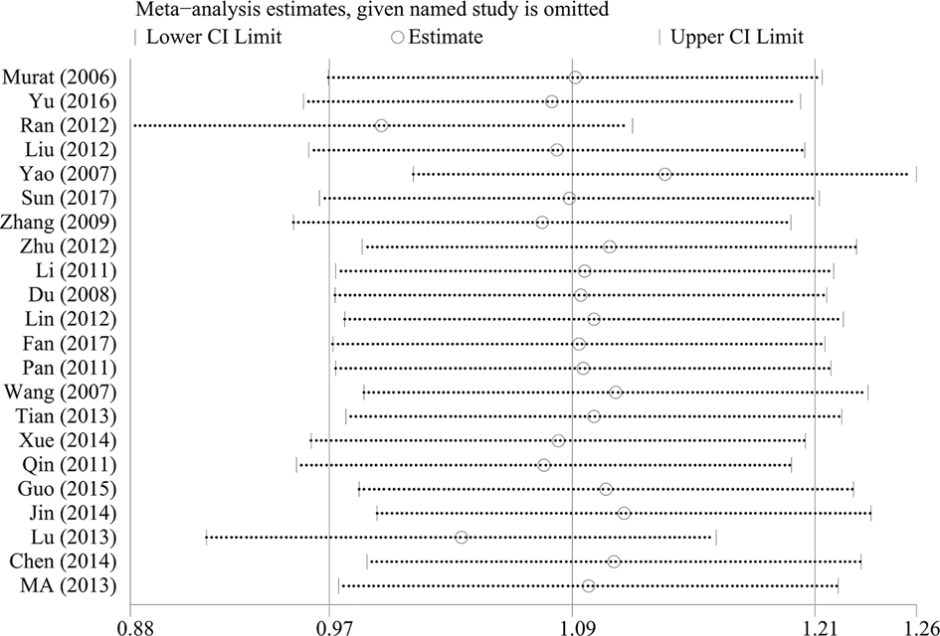
Sensitivity analysis of pooled results for albumin

**Figure 6:**
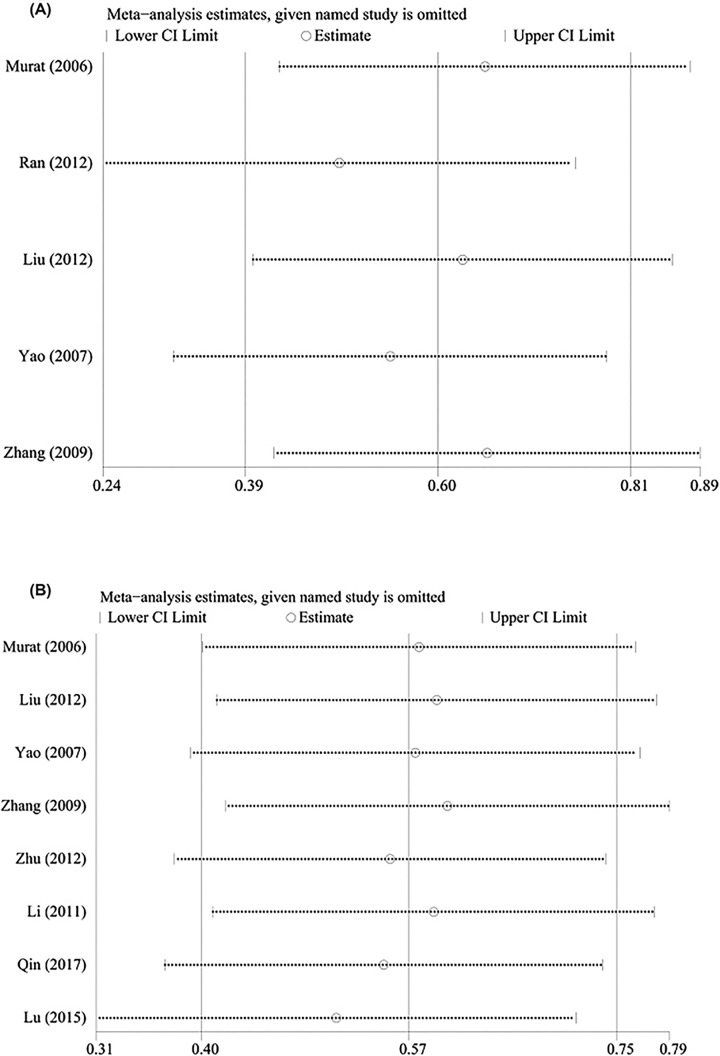
Sensitivity analysis of pooled results Sensitivity analysis of pooled results for total protein (**A**) and transferrin (**B**)

**Figure 7:**
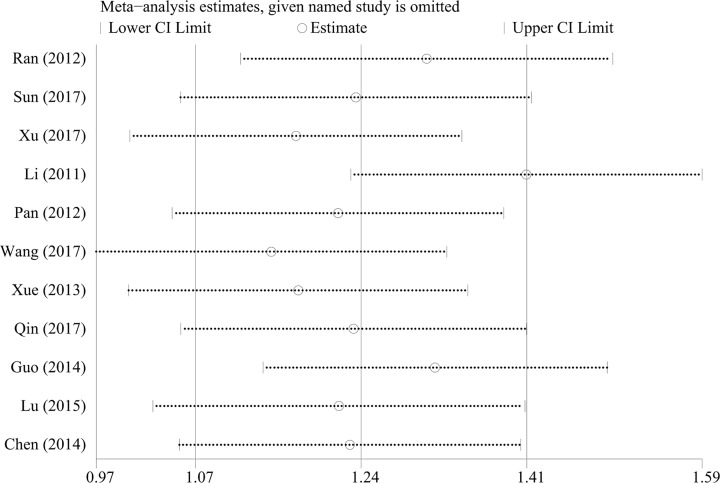
Sensitivity analysis of pooled results for prealbumin

A sensitivity analysis was performed to evaluate the stability of the pooled results. When a study was excluded, the results of the remaining studies were pooled. The results of the sensitivity analysis are presented in Figures 5–7. There was not always a significant change whenever a study was excluded and the results were found to be stable. We performed the Begg's and Egger's test to detect publication bias. No publication bias existed with respect to the results of albumin (Egger: *P* = 0.282, Begg: *P* = 0.338), total protein (Egger: *P* = 0.108, Begg: *P* = 0.213), transferrin (Egger: *P* = 0.639, Begg: *P* = 0.462), and prealbumin (Egger: *P* = 0.082, Begg: *P* = 0.087) levels.

## Discussion

Our meta-analysis from a comprehensive and systematic literature retrieval indicated that (1) L-carnitine elevated the albumin levels in patients on MHD, (2) the total protein and transferrin levels were significantly increased following L-carnitine treatment as compared to the control group, and the prealbumin level was also improved, and (3) subgroup and sensitivity analyses produced similar results. The use of L-carnitine seemed to be useful for improving malnutrition in patients on MHD.

Although the survival rate of patients on MHD improved more significantly than before, the mortality rate of patients on dialysis was still high. Among the numerous affected variables, hypoproteinemia and malnutrition are closely related to mortality in patients on MHD [25]. Malnutrition in MHD patients is associated with complications, such as hyperparathyroidism, anemia, acidosis, etc. Serum albumin, prealbumin, transferrin, total protein levels, and other biochemical indicators are commonly used to evaluate malnutrition status in MHD patients [26]. Our results indicated that L-CN could significantly improve the nutrition status in patients on MHD. The albumin, total protein, transferrin, and prealbumin levels in the trial group were higher than those in the control group. A previous study assessed the effects of L-carnitine on the nutrition status in patients on MHD [27]. However, the previous study was different from our present study in several aspects. First, the focus of attention in the previous study was inflammation, oxidative stress, anemia, dyslipidemia, hyperparathyroidism status, and quality of life, and only five studies were included for estimating the albumin level. On the other hand, our attention was on malnutrition, and hence, we estimated the levels of albumin, total protein, transferrin, and prealbumin. Second, the present study included a higher number of studies. More importantly, our results are totally in contrast with the previous findings that L-carnitine did not improve the nutrition status in MHD patients, while our results indicated that L-carnitine increased the albumin level. Thus, our results have reversed the previous findings.

L-carnitine is a type of water-soluble amino acids that could carry long-chain fatty acids into the mitochondria to participate in reactions and provide energy as a form of adenosine triphosphate [28]. Due to inadequate food intake, nausea, vomiting, poor digestion and absorption, reduced synthesis, and loss in dialysis, MHD patients always have L-carnitine deficiency. Loss of L-carnitine can affect the oxidation of free fatty acids in the mitochondria resulting in the aggregation of lipids in the cytoplasm instead of entering the citric acid cycle [29]. This aggregation leads to a lack of energy. At the same time, acetyl coenzyme A accumulates within the mitochondria and produces cytotoxic effects manifesting as skeletal muscle disease, cardiomyopathy, arrhythmia, and dyslipidemia. Acetyl coenzyme can aggravate malnutrition A by accumulating within the mitochondria [30]. Clinically, nausea, loss of appetite, muscle cramp, cardiac arrhythmia, and hypotension lower the tolerance in patients on dialysis and seriously impact the quality of life, as well as survival [31]. Due to the gastrointestinal response caused by toxins and the restriction of dietary protein, the nutritional status of MHD patients is relatively poor, with moderate malnutrition accounting to about one-third and severe malnutrition accounting for about 6−8% of the total number of patients. With the prolongation of dialysis and an increase in age, the incidence of malnutrition increases, which is an important factor affecting the incidence and mortality of cardiovascular diseases in these patients. A multicenter review revealed that the correlation between malnutrition and mortality in dialysis patients was significantly higher than the dialysis adequacy index [32]. It is reported that the absence of L-carnitine is an important factor causing or aggravating malnutrition in MHD patients. L-carnitine is a key substance required for fatty acid and energy metabolism, and adequate supplementation of L-carnitine can improve the nutritional status of these patients [33]. At present, hematological, and biochemical parameters, such as hemoglobin, and serum levels of total protein, albumin, preprotein, and transferrin are commonly used to clinically evaluate the nutritional status [34]. Our results indicated that these three parameters were significantly improved after L-carnitine therapy. Studies by Wu and Liu found that after 3 months of L-carnitine treatment, the symptoms and signs of patients on MHD in the treatment group were reduced; the hemoglobin level, and blood albumin and transferrin levels were significantly increased; and the incidence of adverse reactions following dialysis was reduced [35,36]. According to the previous studies, the weight, upper arm circumference, thigh circumference, and other anthropometric indicators in dialysis patients were increased after L-carnitine supplementation, and the nutritional status and quality of life of the patients were significantly improved. The results showed that after L-carnitine treatment, the hemoglobin, total protein, and albumin levels in the treatment group were significantly increased as compared to those before treatment and in the control group [28]. This finding is consistent with those of ours. In addition, in terms of the anthropometric measurements, the results of this study showed that the dry body mass and the upper arm muscle circumference in the treatment group were significantly increased as compared to those in the control and treatment groups. The hemoglobin level, total protein level, albumin level, body weight, and upper arm muscle circumference were increased suggesting that the nutritional status of the patients was improved. This may be related to L-carnitine therapy facilitating the oxidation of fatty acids and inhibition of protein decomposition, which is consistent with the findings of Ahmad et al. [37]. However, the present study provided more evidence.

Our study has several limitations. First, the sample size of the included studies was small, and some studies did follow the double-blinding technique. Of the 27 studies included in the meta-analysis, 22 are from China, and it may be associated with the increase of patient's number. None of the included non-Chinese studies are within the last 10 years. The results should be cautiously explained in the other population setting. Second, the heterogeneity within the studies reporting albumin level was high. This heterogeneity might be explained by differences in the baseline albumin level, the dose of L-carnitine, or the study duration. Third, most of the included studies reported short-term (less than one year) outcomes following L-carnitine supplementation, and improving the nutritional parameters will not necessarily result in improved morbidity and mortality. The long-term efficacy of L-carnitine needs to be investigated by further long-term studies. Finally, our study did not include the patients on the peritoneal dialysis. The other outcomes, such as inflammation, dyslipidemia, and hyperparathyroidism should also be focused. Further research is required to address this issue.

In conclusion, the present meta-analysis indicated that L-carnitine has a favorable effect on malnutrition biomarkers in patients on MHD, including the increase in the levels of albumin, total protein, transferrin, and prealbumin. The L-carnitine could be an option for treatment of MHD patients. However, since the selected markers are surrogate ones and not endpoints, the conclusion of this study needs to be further verified by high-quality randomized controlled trials.

## Supplementary Material

Supplementary materials S1-S4

## Abbreviations

Alb: albumin
BMI: body mass index
CI: confidence interval
CKD: chronic kidney disease
L-CN: Levo-carnitine
MHD: maintenance hemodialysis
PA: prealbumin
PEW: protein-energy wasting
SMD: standardized mean difference
TP: total protein
TRF: transferrin
WMD: weighted mean difference
