# Supplementary material for: The efficacy of L-carnitine in improving malnutrition in patients on maintenance hemodialysis: a meta-analysis
Source: Biosci Rep. 2020 Jun 15;40(6):BSR20201639. doi: 10.1042/BSR20201639 (PMC7295622; doi:10.1042/BSR20201639)
Supplement: Supplementary materials S1-S4 [file BSR-2020-1639_supp.pdf]

Search strategy

(1) PubMed

Step 1.carnitine[MeSH]OR carnitine *[MeSH]OR L-carnitine *[MeSH]OR Levcarnitine*[MeSH b]

Step 2. maintenance hemodialysis [MeSH]OR hemodialysis [MeSH]OR MHD [MeSH b]

Step 3.malnutrition [MeSH]OR nutrition [MeSH]

Step 4.randomized controlled trial[MeSH]OR controlled clinical trial [MeSH]OR randomizedMeSH]OR controlled[MeSH]OR trial[MeSH]OR random[MeSH]OR placebo [MeSH]OR groups[MeSH] OR cohort study OR cohort OR perspective OR retrospective OR follow-up

Step 5: Step 1 AND Step 2 AND Step 3 AND Step 4

Step 6 animals [MeSH] not humans [MeSH]

Step 7:Step 1 AND Step 2 AND Step 3 AND Step 4 not Step 6.

(2) Web of Science AND Embase

1TS=(malnutrition) AND TS=(hemodialysis)Indexes=SCI-EXPANDED, SSCI, A&HCI, CPCI-S, CPCI-SSH, ESCI, CCR-EXPANDED, IC Timespan=All years

2 TOPIC: (L-carnitine) OR TOPIC: (Levcarnitin) OR TOPIC: (carnitine) OR TOPIC: (carnitine)Indexes=SCI-EXPANDED, SSCI, A&HCI, CPCI-S, CPCI-SSH, ESCI, CCR-EXPANDED, IC Timespan=All years

#2 AND #1Indexes=SCI-EXPANDED, SSCI, A&HCI, CPCI-S, CPCI-SSH, ESCI, CCR-EXPANDED, IC Timespan=All years

(3) Chinese Database (CNKI and Wangfang)

Step 1: 左卡尼汀 OR 卡尼汀 OR 左旋肉毒碱

Step 2: MHD OR 维持性血液透析

Step 3: 营养不良 OR 营养

Step 4: RCT OR 随机对照试验 OR 临床试验 OR 队列研究

Step 5: Step 1 AND Step 2 AND Step 3 AND Step 4

References

1. Zhang SX, Song XD, Zhao DT: The therapeutic effect of levocarnitine in maintenance hemodialysis patients. Chin Mod Doctor, 2009; 47：94-95.

2. Liu H: The significance of levocarnitine in maintenance hemodialysis. Guide China Med, 2012; 10：146-47.

3. Li L: Observation of levocarnitine on malnutrition and quality of life improvement in hemodialysis patients. Strait Pharm J, 2011; 23：137-38.

4. Tian QS: Efficacy of levocarnitine in maintenance hemodialysis patients. Jiangsu Med J, 2011; 37：821-22.

5. Guo J, Yuan L, Chen YH, Xie P et al: Effects of levocarnitine on T lymphocyte subsets and nutritional status in maintenance hemodialysis patients. Shanghai Med, 2014; 37：502-06.

6. Yu WZ, Gao GS: Effects of levocarnitine on nutritional parameters and inflammatory status in maintenance hemodialysis patients. Hua Med, 2016; 31：25-28.

7. Ma H: Effects of levocarnitine on nutritional and microinflammatory status in maintenance hemodialysis patients. Guangxi Med, 2013; 35：1632-34.

8. Wang JL: Effects of levocarnitine on microinflammatory status and nutritional status in maintenance hemodialysis patients. J Benbu Med, 2017; 42：1692-95.

9. Fan YR, Qu KY: Effects of levocarnitine on malnutrition and microinflammatory status in maintenance hemodialysis patients. J Commun Med, 2009; 7：11-12.

10. Xue HY: Effects of levocarnitine on inflammation, malnutrition and anemia in elderly patients undergoing maintenance hemodialysis. Shanxi Med, 2013; 42：1611-12.

11. Qin X, Chen W: Effects of levocarnitine on inflammation and nutritional status in elderly

patients with end-stage renal disease on maintenance hemodialysis. Chin J Gerontol, 2017; 37：4880-82.

12. Sun XL: Effects of levocarnitine combined with hemodialysis on nutritional status and quality of life in elderly patients with maintenance hemodialysis. Chron Pathematol J, 2017; 18：294-96.

13. Lin Y, Lin XC: Effects of levocarnitine adjuvant therapy on serum nutritional indexes and inflammatory cytokines levels in patients with maintenance hemodialysis. China Pharm, 2015; 18：1914-15.

14. Duranay M, Akay H, Yilmaz FM, Senes M et al: Effects of L-carnitine infusions on inflammatory and nutritional markers in haemodialysis patients. Nephrol Dial Transplant, 2006; 21：3211-14.

15. L J: Effect of levocarnitine on nutritional status of maintenance hemodialysis patients in plateau area. Chin J Integr Trad&West Neph, 2011; 12：916.

16. Pan YQ: Effect of levocarnitine on nutritional status of maintenance hemodialysis patients. Jilin Med, 2012; 33：4324-25.

17. Ran YL, Han L, Tan H: Effect of levocarnitine on malnutrition and heart failure in elderly patients receiving hemodialysis. Med J Chin Peop Arm Pol For, 2012; 23：147-49.

18. Yao L, Zhang XM, Zhang YM, Zhang XZ et al: Clinical use of L-carnitine supplementation in maintenance hemodialysis patients. J Clin Intern Med, 2007; 24：694-96.

19. Chen DG, Hu YL, Hu QD: Clinical study of levocarnitine improving malnutrition and microinflammation state of maintenance hemodialytic patients. J Luzhou Med College, 2014; 37：427-29.

20. Zhu FL. Clinical effects of levocarnitine on maintenance hemodialysis patients [Harbin: Jilin University; 2012.

21. Xu HP, Ma XY, Hu J, Li XL: Clinical effect of levonidine in the treatment of malnutrition caused by maintenance hemodialysis. Chin J Clin Rational Drug Use, 2017; 10：70-71.

22. Lu JY, Shi DY, Yu C: A two-center study on the effects of levocarnitine on inflammatory factors, malnutrition and anemia in patients undergoing maintenance hemodialysis. J Hainan Med College, 2015; 21：65-67.

23. Ahmad S, Robertson HT, Golper TA, Wolfson M et al: Multicenter trial of L-carnitine in maintenance hemodialysis patients. II. Clinical and biochemical effects. Kidney Int, 1990; 38：912-18.

24. Biolo G, Stulle M, Bianco F, Mengozzi G et al: Insulin action on glucose and protein metabolism during L-carnitine supplementation in maintenance haemodialysis patients. Nephrol Dial Transplant, 2008; 23：991-97.

25. Duranay M, Akay H, Yilmaz FM, Senes M et al: Effects of L-carnitine infusions on inflammatory and nutritional markers in haemodialysis patients. Nephrol Dial Transplant, 2006; 21：3211-14.

26. Savica V, Santoro D, Mazzaglia G, Ciolino F et al: L-carnitine infusions may suppress serum C-reactive protein and improve nutritional status in maintenance hemodialysis patients. J Ren Nutr, 2005; 15：225-30.

27. Steiber AL, Davis AT, Spry L, Strong J et al: Carnitine treatment improved quality-of-life measure in a sample of Midwestern hemodialysis patients. JPEN J Parenter Enteral Nutr, 2006; 30：10-15.

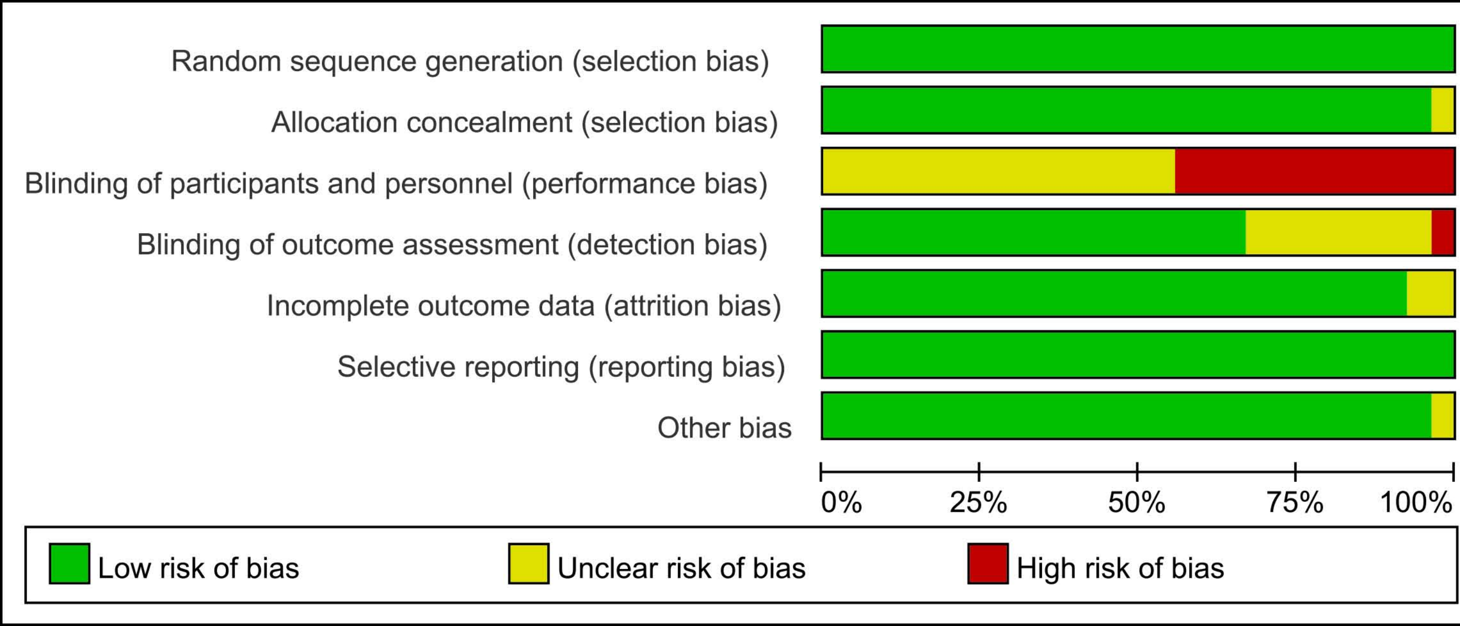
Random sequence generation (selection bias)
Allocation concealment (selection bias)
Blinding of participants and personnel (performance bias)
Blinding of outcome assessment (detection bias)
Incomplete outcome data (attrition bias)
Selective reporting (reporting bias)
Other bias
0%
25%
50%
75%
100%
Low risk of bias
Unclear risk of bias
High risk of bias

Supplementary material Figure S3: Risk of bias and applicability concerns graph: review authors' judgements about each domain presented as percentages across included studies

	Random sequence generation (selection bias)	Allocation concealment (selection bias)	Blinding of participants and personnel (performance bias)	Blinding of outcome assessment (detection bias)	Incomplete outcome data (attrition bias)	Selective reporting (reporting bias)	Other bias
Ahmad 1990	+	+	−	−	+	+	+
Biolo 2008	+	+	?	+	+	+	+
Chen 2014	+	+	−	?	+	+	+
Duranay 2006	+	+	−	?	+	+	+
Fan 2009	+	?	−	+	+	+	?
Guo 2014	+	+	?	+	+	+	+
Jin 2011	+	+	?	+	+	+	+
Li 2012	+	+	−	+	+	+	+
Lin 2015	+	+	−	+	+	+	+
Liu 2012	+	+	−	+	?	+	+
Lu 2014	+	+	?	?	+	+	+
Ma 2013	+	+	?	?	+	+	+
Murat 2006	+	+	?	+	+	+	+
Pan 2012	+	+	?	+	+	+	+
Qin 2014	+	+	−	+	+	+	+
Ran 2012	+	+	?	?	+	+	+
Savica 2005	+	+	−	+	+	+	+
Steiber 2006	+	+	?	?	+	+	+
Sun 2017	+	+	?	+	?	+	+
Tian 2011	+	+	?	?	+	+	+
Wang 2017	+	+	?	+	+	+	+
Xu 2017	+	+	?	+	+	+	+
Xue 2013	+	+	?	+	+	+	+
Yao 2007	+	+	−	?	+	+	+
Yu 2016	+	+	−	+	+	+	+
Zhang 2009	+	+	?	+	+	+	+
Zhu 2012	+	+	−	+	+	+	+

Supplementary material Figure S4: Risk of bias and applicability concerns summary: review authors' judgements about each domain for each included study
